# At Last

**Published:** 1899-07

**Authors:** 


### At Last.

The volume of Transactions of the National Dental Association, including the minutes of the convention by which the National Dental Association was organized, and of the first meeting of the proceedings of the second annual session held at Omaha, together with the proceedings of the first annual session of the Southern Dental Association, has just come to hand.

We are glad to welcome the volume, but much regret that it did not appear six or eight months ago. Many of the members of the Association have suffered great inconvenience because of its delay. The volume is well gotten up, while that is gratifying it does not compensate for the delay of its issue.

It is greatly desired that the publication committee will give more special attention to this work in the future than hitherto. If the Association is to prosper the proceedings should be made available to the profession, at least to the membership, as early as possible after the meeting is held. The Association can not afford to repeat a delay of this kind very often. We hope for better things in the future.

